# New Echocardiographic Parameters Predicting Successful Trans-Ventricular Beating-Heart Mitral Valve Repair with Neochordae at 3 Years: Monocentric Retrospective Study

**DOI:** 10.3390/jcm12051748

**Published:** 2023-02-22

**Authors:** Alessandro Vairo, Lorenzo Gaiero, Matteo Marro, Caterina Russo, Marco Bolognesi, Paolo Soro, Guglielmo Gallone, Francesco Fioravanti, Paolo Desalvo, Fabrizio D’Ascenzo, Gianluca Alunni, Viviana Sebastiano, Cristina Barbero, Marco Pocar, Gaetano Maria De Ferrari, Mauro Rinaldi, Stefano Salizzoni

**Affiliations:** 1 Division of Cardiology, Cardiovascular and Thoracic Department, Città della Salute e della Scienza Hospital, 10126 Turin, Italy; 2 Division of Cardiac Surgery, Department of Surgical Sciences, Città della Salute e della Scienza di Torino, University of Turin, 10126 Turin, Italy; 3 Department of Medical Sciences, University of Turin, 10126 Turin, Italy; 4 Department of Clinical Sciences and Community Health, University of Milan, 20122 Milan, Italy

**Keywords:** mitral valve prolapse, mitral annulus disfunction, three-dimensional echocardiography, micro-invasive cardiac surgery

## Abstract

The NeoChord procedure is an echo-guided trans-ventricular beating-heart mitral valve repair technique to treat degenerative mitral regurgitation (MR) due to prolapse and/or flail. The aim of this study is to analyze echocardiographic images to find pre-operative parameters to predict procedural success (≤moderate MR) at 3-year follow-up. Seventy-two consecutive patients with severe MR underwent the NeoChord procedure between 2015 and 2021. MV pre-operative morphological parameters were assessed using 3D transesophageal echocardiography with dedicated software (QLAB, Philips). Three patients died during their hospitalization. The remaining 69 patients were retrospectively analyzed. At follow-up, MR > moderate was found in 17 patients (24.6%). In the univariate analysis, end-systolic annulus area (12.5 ± 2.5 vs. 14.1 ± 2.6 cm^2^; *p* = 0.038), end-systolic annulus circumference (13.2 ± 1.2 vs. 14 ± 1.3 cm; *p* = 0.042), indexed left atrial volume (59 ± 17 vs. 76 ± 7 mL/m^2^; *p* = 0.041), and AF (25% vs. 53%; *p* = 0.042) were lower in the 52 patients with ≤ MR compared to those with > moderate MR. Annular dysfunction parameters were the best predictors of procedural success: 3D early-systolic annulus area (AUC 0.74; *p* = 0.004), 3D early-systolic annulus circumference (AUC 0.75; *p* = 0.003), and 3D annulus area fractional change (AUC 0.73; *p* = 0.035). Patient selection relying on 3D dynamic and static MA dimensions may improve the maintenance of procedural success at follow-up.

## 1. Introduction

Degenerative MR is one of the most common valvular heart diseases requiring surgery in the West [1]. In the last few years, many techniques and devices have been introduced and used for mitral valve repair (MVR) to avoid extracorporeal circulation and reduce perioperative complications [2]. Trans-ventricular beating-heart MVR (TBMVR) by artificial chordae implantation has been demonstrated to be safe and feasible [3]. This echo-guided procedure had good clinical outcomes at mid-term follow-up, but patient selection played an essential role in the repair’s durability [4]. Three-dimensional (3D) echocardiography has already been described as a fundamental tool to direct this beating-heart repair in terms of chordal implantation position and morphological analysis after neochordae traction and fixation [5,6]. A pre-procedural echocardiographic analysis of the entire mitral valve apparatus has been an additional critical feature to identify patients who may benefit from a ringless MVR. In this monocentric and retrospective study, we investigated the mid-term results of TBMVR. This study has demonstrated that mitral annulus 3D echocardiographic features could predict the success and stability of the procedure at follow-up.

## 2. Materials and Methods

Between March 2015 and February 2021, 72 consecutive patients with severe MR due to prolapse and/or flail, who underwent MV repair with the NeoChord DS1000 system (NeoChord, Inc., Eden Prairie, MN, USA) at the Città della Salute e della Scienza Hospital in Turin, were included in this retrospective evaluation. After assessment of eligibility for the Neochord procedure, patients were selected considering the following characteristics [7,8,9]:

– Thirty-one patients were chosen because they were over the age of 80 years.
– Fourteen patients had a recent or ongoing malignancy.
– Six patients had contraindications to a standard right thoracotomy.
– Six patients suffered from severe lung disease and six patients had previous cardiac surgery.
– Five patients had severe liver disease.
– In accordance with their wishes, four patients underwent NeoChord.

All procedures were performed by the same operator, who, after his learning curve, supervised or taught a second surgeon. All patients underwent preoperative transesophageal echocardiography (TEE) at our Cardiology Division to establish the grade and mechanism of MR. The iE33 and EPIQc7 ultrasonography systems, equipped with X7-2T and X8-2T transducers (Philips Healthcare), were used to acquire 3D TEE data. Anatomical classification was applied to describe the MV morphology as follows:

– “Type A”, isolated central posterior mitral leaflet (PML) disease
– “Type B”, PML (lateral, medial, or posterior) multi-segment disease
– “Type C”, anterior mitral leaflet (AML) disease or bi-leaflet disease

Only patients with fibro-elastic disease and a leaflet-to-annulus index (LAI) ≥ 1.2 were included [10]. Patients with endocarditis, functional MR, congenital malformations of MR, and Barlow disease were excluded from enrollment. Annular dilatation is identified when the ratio of annulus diameter/anterior leaflet length is >1.3 (in diastole) or when the antero-posterior annulus diameter is >35 mm. Commissural diameter can be measured in the TTE apical two-chamber view or in the TOE at 50°. The normal contraction of the mitral annulus (decrease in annular area in systole) is 25% [11]. In addition to standard 2D echocardiographic measurements, the researchers calculated early-systolic, end-systolic, end-diastolic mitral annulus (MA) area and circumferences, end-systolic annulus height, leaflet area, and anatomic regurgitant orifice in a post-processing workstation using commercially available software (QLAB, Philips Health-Care). After the procedure, patients underwent clinical and echocardiographic follow-up at discharge, then after one, three, six, and twelve months, and yearly thereafter. MR severity was graded as absent or trace, mild, moderate, and severe according to the American Society of Echocardiography criteria [12]. Outcomes were defined according to the Mitral Valve Academic Research Consortium guidelines [13]. Procedure success, assessed at follow-up, was defined as freedom from more than moderate MR, from rehospitalizations or reintervention for the underlying condition (e.g., MR or heart failure), and improvement from baseline in symptoms estimated with the NYHA functional class. Starting from these data, we submitted MV 3D parameters to statistical analysis to evaluate their predictive ability of outcome in terms of MR reduction. Categorical variables are presented as percentages and continuous variables as mean ± SD or median [IQR]. Statistical Package for Social Sciences (SPSS Inc., Chicago, IL, USA) was used for statistical analyses. A Cox regression analysis was performed, including all variables with a *p* < 0.05 at univariate analysis and with less than 20% of missing data. Each variable that presented a significant predictive value at univariate analysis was analyzed with a ROC curve. The ability of the ROC curves to predict the consistently good results of the procedure events was measured with the Area Under the Curve (AUC). An AUC of 0.6 or below was considered poor, an AUC of 1.0 was considered excellent, and an AUC of 0.8 was considered good.

## 3. Results

### 3.1. Procedural Data and Follow-Up

Patient characteristics and echocardiographic parameters of the population analyzed are summarized in Table 1 and Table 2.

A median of three neochords were implanted for each procedure. There was no conversion to open surgery; no mechanical circulatory support was needed; and there were no intraoperative deaths. Three in-hospital deaths occurred. One patient was discharged with moderate-to-severe MR (only one chord was implanted for technical issues) and did not undergo further intervention due to very high-risk general conditions. Procedural success at discharge was therefore achieved in sixty-eight (94.5%) patients (Table 3).

**Figure 1:**
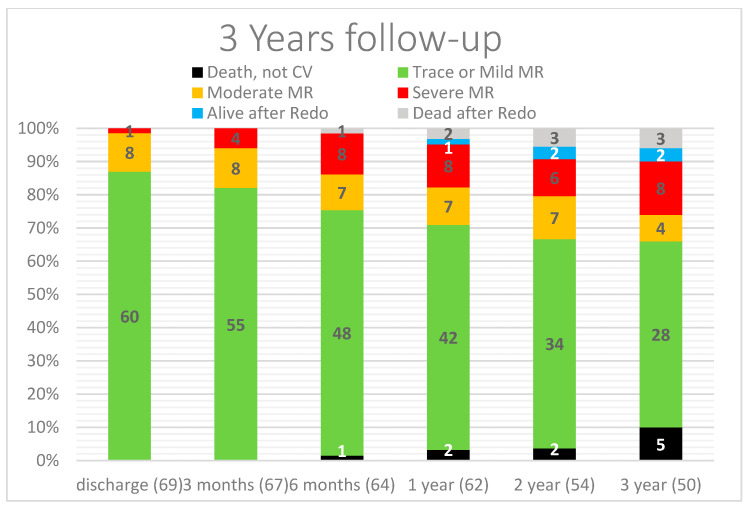
Outcomes at 3-year follow-up. CV: cardiovascular; MR: mitral regurgitation.

Sixty-nine patients were observed from discharge with a median follow-up of 34 months (IQR: 12–36 months) and represent the study population: 52 patients had moderate or less MR (success group); 17 patients > moderate MR (failed group). Follow-up visits at three months, six months, one year, two years, and three years were performed for 97%, 94%, 89%, 78%, and 73% of patients, respectively. At three-year follow-up, 4 patients (4/69 = 6%) had moderate-to-severe MR, and 13 patients (19%) presented with severe MR. Of the latest, five patients (7%) were reoperated; two of them survived and three died of hospitalization-related complications. Eight patients (12%) did not undergo surgery after careful evaluation of the risk-benefit ratio by the Heart Team. Examining the 17 failed patients at a three-year follow-up, the main reason for MR recurrence was found to be elongation/detention of the artificial chordae in eight patients, caused by reverse ventricular remodeling. The other mechanisms found by TTE and TEE were rupture of native chordae, neochord detachment, and annular dilatation. Table 4 summarizes the causes of MR recurrence and their timing. Excluding patients that died or that required a new surgical operation, the prevalence of mild or trace MR was 82% at 3 months, 76% at 6 months, 74% at 1 year, 72% at 2 years, and 70% at 3 years [Figure 1].

At the univariate analysis, none of the pre-operative characteristics in Table 1 were statistically different, except for the presence of AF: 13/52 (25%) in the success group vs. 9/17 (53%) in the failed group (*p* = 0.042). This characteristic remains significative also at univariate regression analysis (OR 3.38, IC 1.08–10.56) as shown in Table 5.

There was no association between the procedure’s date and outcome in the logistic regression analysis [HR for increasing year (2015 reference): HR 1.00, 95%CI 0.99–1.01, *p* = 0.446].

### 3.2. Echocardiographic Predicting Variables

Pre-operative MV parameters were divided into four groups to investigate the potential correlation with absent or mild MR at follow-up: leaflets; coaptation reserve; static and dynamic MA parameters. Baseline echocardiographic measures at last follow-up in the two groups are shown in Table 6.

**Figure 2:**
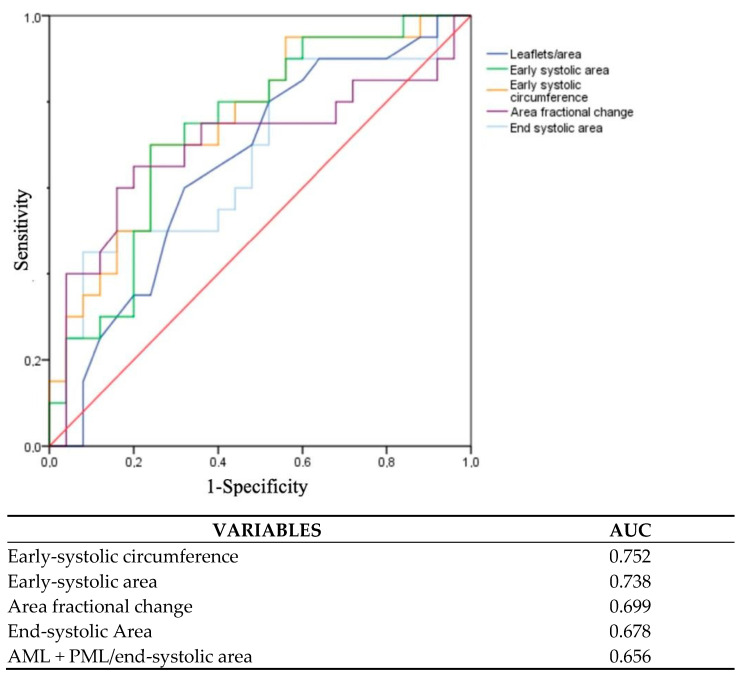
ROC and AUC of variables with best predictive values for moderate or less mitral regurgitation at last follow-up. AML: anterior mitral leaflet; AUC: area under the curve; PML: posterior mitral leaflet. Red line in the graph: test neutrality line.

The most significant results were found analyzing annular dysfunction through 3D MA dynamic parameters: pre-operative early-systolic MA area (12.5 ± 2.5 vs. 14.1 ± 2.6 cm^2^; *p* = 0.038) and MA circumferences (13.2 ± 1.2 vs. 14 ± 1.3 cm; *p* = 0.042) were strongly associated with success procedure at last follow-up visit in patients undergoing TBMVR procedure. Receiver-operating characteristic curves of variables that presented a significant predictive value at univariate analysis are shown in Figure 2.

Upon curve analysis, the most accurate parameter in predicting a successful procedure at the last follow-up was the MA disfunction parameter defined with 3D techniques; in particular, the area under the curve was greatest for early-systolic circumferences ([AUC] 0.752) and early-systolic area ([AUC] 0.738).

All preoperative parameters with a *p* < 0.05 in the univariate analysis were also tested with a multivariate analysis, but none of the parameters was statistically significant, realistically due to the relatively small sample size.

## 4. Discussion

In the field of micro-invasive cardiac surgery (off-pump procedures through a small incision), TBMVR with artificial chordae implantation represents one of the most developed and promising technologies for degenerative MR [2]. Our study confirmed the safety and feasibility of this procedure, even in an elderly population with comorbidities. D’Onofrio et al. showed that mortality and complication rates were comparable to conventional cardiac surgery [14]. The ability to select the most appropriate patients for this procedure has increased over time. Initially, anatomical type was considered the most important predictive parameter [15]. D’Onofrio et al. demonstrated that an appropriate anatomical classification (type A, B, or C) and the leaflet-to-annulus index (LAI) ≥ 1.25 were mandatory to improve the chances of success [16]. In fact, Colli proved that excess leaflet tissue in proportion to annular dimension increases the predicted coaptation surface after the procedure. 

In the current study, LAI was not significant but cannot be reliably determined because patients were already selected with LAI > 1.2. This, on the other hand, has demonstrated that LAI cannot be the only parameter to predict a good outcome after Neochord. This was also demonstrated by a nomogram combining seven variables (anatomical and echocardiographic) that has been introduced to predict the probability of mild or less residual MR at follow-up periods [17].

This study suggested that quantification of MA remodeling could be an additional promising parameter to predict a successful MV repair without reductive annuloplasty. Many studies with in vivo 3D echocardiography and an ex vivo heart simulator proved that the dynamic nature of annulus change was fundamental for efficient function of the MV, reducing systolic strains of the posterior leaflet and peak leaflet stress [18,19]. Early-systolic contraction of the MA area has been observed in both healthy subjects and patients with degenerative MR valves, followed by gradual MA area increase until the end of systole. Instead, in some dysfunctional MVs (i.e., ischemic and myxomatous; atrial fibrillation), the MV annulus appeared less dynamic, and the MA area contracted less during early systole (mean annular fractional change, 19% vs. 10%) [20,21]. Based on these assumptions and on the documented relation between the presence of AF and enlarged index left atrial volume and the worst outcomes in terms of residual MR at follow-up, a decision to look for a link between preoperative MA function (through the analysis of dynamic three-dimensional measurements with dedicated software) and MVR outcome was made. A good correlation, particularly with the early-systolic area and circumference, was demonstrated. This result has not been surprising, as early systole is the phase in the cardiac cycle when the peak of annulus contraction is reached and when the greatest discrepancy of annulus area has been shown between healthy people and patients with mitral valve disease. Furthermore, annular dilatation combined with the loss of its saddle shape increased traction on leaflets and could explain the mechanism of some failures that were observed. Stabilization after reverse remodeling was less permissive in this context, and this, combined with increased stress on the MV apparatus, promoting native ruptures, elongation, and detachment, could result in a new loss of coaptation and recurrence. This result suggested that valves with a better contracting and less dilated annulus may have better outcomes in terms of residual regurgitation, particularly in the field of MVR without annuloplasty. Definitely, it has introduced a new dynamic pre-operative echocardiographic predictive parameter for the first time.

These data were also supported by the logistic regression analysis. Indeed, there was no association between the date of the procedure and the outcome, indicating that there was no surgical bias. This has emphasized the need for careful patient selection based on pre-operative anatomy and function of the mitral valve apparatus. Moreover, as Narang et al. demonstrated, reduced compressive circumferential strain was associated with worse results of surgical ischemic MVR with annuloplasty [22], and it was proposed as a method that could help optimize reparation technique to reduce post-surgical MR recurrence. It would be interesting to transpose this advanced quantification approach into the field of degenerative MVR.

The transcatheter edge-to-edge technique was not considered for this population, even if a recent paper by Benfari et al. shows acceptable results in this kind of population [23].

We believe that a surgical-like result is more appropriate and physiological when feasible. Indeed, the placement of neochords leaves no bulky leaflet clip devices and may allow for additional surgical and/or transcatheter procedures in the future; in particular, it does not preclude a new attempt at MVR.

Recently, Latib et al. performed the first case of trans-septal MVR with the Neochord NeXuS system, reporting a successful three-month follow-up [24]. This technique represents the natural evolution after more than ten years of TBMVR but needs further data before replacing the trans-ventricular approach. Furthermore, papillary muscle position and size may be a limitation for patient selection. It is therefore conceivable to assume that the trans-ventricular approach, also considering its role in aortic disease, could still be considered.

## 5. Conclusions

TBMVR with artificial chordae implantation is a micro-invasive technique with a good therapeutic success and safety profile. Patient selection relying on dynamic and static mitral annulus dimensions through 3D analysis may improve the successful outcome of the procedure at follow-up. Further studies are needed to optimize the selection of candidates and the reproducibility of these results regarding the predictive value of annular dysfunction in ringless mitral valve repair, which could also have implications for all other mitral surgery techniques.

## Figures and Tables

**Table 1:** Baseline demographic and clinical characteristics.

Patient Characteristics n = 72	Mean ± SD n (%)
Age (years)	77 ± 9
Female	27 (37.5%)
EuroSCORE II (%)	2.2 ± 1.5
eGFR (mL/min)	65 ± 22
NYHA ≥ III	63 (87.5%)
DM II	4 (5.5%)
Arterial hypertension	45 (62.5%)
Extracardiac Arteriopathy	6 (8.5%)
CAD	12 (16.5%)
Previous Stroke	2 (2.7%)
COPD	5 (7%)
Malignancy	14 (19.5%)
Atrial Fibrillation	23 (32%)
Previous cardiac surgery	6 (8.5%)

eGFR: estimated glomerular filtration rate; NYHA: New York Heart Association functional class; DM II: type 2 diabetes mellitus; CAD: coronary artery disease; COPD: chronic obstructive pulmonary disease.

**Table 2:** Baseline echocardiographic parameters.

Echocardiographic Characteristics	Mean ± SD
Ejection Fraction (%)	63 ± 5
LVEDD (mm)	54 ± 6
EDVi (mL/m^2^)	73 ± 21
ESVi (mL/m^2^)	26 ± 7
Left atrium volume (mL/m^2^)	66 ± 27
sPAP (mmHg)	44 ± 15
AP annulus diameter 2D (mm)	33.9 ± 5.2
IC annulus diameter 2D (mm)	39 ± 4.9
AP annulus diameter 3D (mm)	34.8 ± 4.5
IC annulus diameter 3D (mm)	44 ± 4
AML length (mm)	26.7 ± 4
PML length (mm)	17.9 ± 3.2
Leaflet-to-annulus index (LAI) 2D	1.28 ± 0.21
Leaflet-to-annulus index (LAI) 3D	1.25 ± 0.21
Flail gap (mm)	5.9 ± 2
Flail width (mm)	13 ± 5
Prolapse area (mm^2^)	211± 88
End-systolic annulus area (mm^2^)	1327 ± 261
Early-systolic annulus area (mm^2^)	1236 ± 279
End-diastolic annulus area (mm^2^)	1394± 268
End-systolic annulus circumference (mm)	135 ± 13
Annulus area fractional chance (%)	13 ± 16
Annulus circumference fractional change (%)	4.2 ± 5.3
Leaflet area (mm^2^)	1590 ± 319
Anatomic regurgitant orifice (mm^2^)	70 ± 34
End-systolic annulus height (mm)	6.1 ± 1.7
**Echocardiographic characteristics**	**n (%)**
Tricuspid regurgitation ≥ moderate	13 (18%)
Aortic Regurgitation ≥ moderate	1 (1.4%)
Aortic stenosis ≥ moderate	0 (0%)
**Anatomical Classification**	
Type A (favorable)	42 (58.3%)
Type B (favorable)	24 (33.3%)
Type C (unfavorable)	6 (8.3%)
Spot annulus calcification	7 (9.7%)
Cleft	4 (5.5%)

AML: anterior mitral leaflet; AP: antero-posterior; EDD: end-diastolic diameter of the left ventricle; EDVi: end-diastolic volume index; ESVi: end-systolic volume index; IC: intercommissural; sPAP: systolic pulmonary artery pressure; PML: posterior mitral leaflet.

**Table 3:** In-hospital data.

Parameter	Median [IQR] or n (%)
NeoChordae implanted (n)	3 [3–4]
<3	2 (2.8%)
3	35 (48.6%)
4	28 (38.9%)
5	5 (6.9%)
>5	2 (2.8%)
Mechanical ventilation time (hours)	5.5 [4–7]
Intensive Care Unit length of stay (days)	1 [1–1]
Conversion to conventional surgery	0
Mechanical Support	0
Transient Ischemic Attack	1 (1.4%)
Stroke	1 (1.4%)
Renal replacement therapy	0
Cardiac tamponade	1 (1.4%)
In-hospital death	3 (4%)
Procedural success at discharge	68 (94.5%)

**Table 4:** Mechanisms of MR recurrence.

Mechanism of MR Recurrence	FU < 1 y (n = 62)	FU 1–3 y (n = 50)	FU ≥ 3 y (n = 40)
NeoChord detension	3	3	2
NeoChord rupture at the base of the LV	2	-	1
MV Annular dilatation	-	-	2
NeoChord detachment from leaflet	1	-	-
Native chords rupture	3	-	-

MR: mitral regurgitation; FU: follow-up; y: year; LV: left ventricle.

**Table 5:** Univariate analysis using descriptive statistics and univariate logistic regression analysis on baseline demographic and clinical characteristics (69 patients, 3-year follow-up, outcome: MR grade).

Parameter	Success Group n = 52	Failed Group n = 17	Univariate *p*-Value	OR	Univariate 95% CI	Logistic Regression *p*-Value
Age (years)	75.1 + 8.3	75.7 + 7.4	0.660	1.01	0.94–1.08	0.777
EuroSCORE II (%)	2.3 + 1.9	2.5 + 2	0.873	1.03	0.77–1.37	0.852
eGFR (mL/min)	64.1 + 20.4	62.2 + 25.2	0.554	1.00	0.97–1.02	0.761
Female	19 (36.5%)	7 (41.2%)	0.778	1.22	0.40–3.72	0.732
NYHA ≥ III	45 (86.5%)	15 (88.2%)	>0.999	1.17	0.22–6.24	0.857
DM II	3 (5.8%)	1 (5.9%)	>0.999	1.02	0.10–10.52	0.986
Arterial hypertension	33 (63.5%)	10 (58.8%)	0.778	0.82	0.27–2.52	0.732
Extracardiac Arteriopathy	4 (7.7%)	1 (5.9%)	>0.999	0.75	0.08–7.21	0.803
CAD	8 (15.4%)	2 (11.8%)	>0.999	0.73	0.14–3.84	0.714
Previous Stroke	1 (1.9%)	0 (0%)	>0.999	---	N/A	---
COPD	3 (5.8%)	2 (11.8%)	0.590	2.18	0.33–14.28	0.417
Malignancy	8 (15.4%)	3 (17.6%)	>0.999	1.18	0.27–5.06	0.825
Atrial Fibrillation	13 (25%)	9 (53%)	0.042	3.38	1.08–10.56	0.037
Previous cardiac surgery	2 (3.8%)	3 (17.6%)	0.092	5.36	0.81–35.28	0.081

OR: odds ratio; CI: confidence interval; MR: mitral regurgitation; eGFR: estimated glomerular filtration rate; NYHA: New York Heart Association functional class; DM II: type 2 diabetes mellitus; CAD: coronary artery disease; COPD: chronic obstructive pulmonary disease.

**Table 6:** Baseline echocardiographic and MV parameters according to residual MR at last follow-up.

Parameter	Success Group n = 52	Failed Group n = 17	*p*-Value
Ejection fraction (%)	63.9 ± 4.5	62.7 ± 6	0.377
LVEDD (mm)	53.7 ± 6.9	54.25 ± 5.8	0.793
EDVi (mL/m^2^)	71 ± 22	77.3 ± 17	0.395
ESVi (mL/m^2^)	25 ± 7	28 ± 7	0.269
Left atrial volume (mL/m^2^)	59 ± 17	76 ± 37	0.041
sPAP (mmHg)	42 ± 13	47 ± 18	0.195
**Leaflets**			
Anatomic regurgitant orifice (mm^2^)	71 ± 34	69 ± 35	0.84
Prolapse area (mm^2^)	206 ± 75	219 ± 10	0.61
AML + PML (mm)	44.4 ± 6.1	43.6 ± 6.3	0.53
Leaflet area (mm^2^)	1672 ± 308	1524 ± 317	0.12
**Annulus, Static**			
AP annulus diameter 2D (mm)	33.77 ± 4.57	34 ± 5.5	0.89
End-systolic annulus height (mm)	6.2 ± 1.8	5.9 ± 1.7	0.54
IC/AP (ellipticity) (mm)	1.27 ± 0.1	1.24 ± 0.1	0.40
IC annulus diameter 2D (mm)	38.2 ± 5.3	39.4 ± 4.8	0.39
IC annulus diameter 3D (mm)	43.2 ± 4.1	45.1 ± 3.7	0.11
AP annulus diameter 3D (mm)	33.8 ± 4.5	36.2 ± 4.2	0.06
[(AP + IC)/2] 3D value	38.7 ± 4	40.8 ± 3	0.06
End-diastolic annulus area (mm^2^)	1326 ± 254	1482 ± 267	0.05
End-diastolic annulus circumference (mm)	135 ± 13	143 ± 12	0.04
End-systolic annulus circumference, (mm)	131.9 ± 12	139.5 ± 12	0.042
End-systolic annulus area (mm^2^)	1257 ± 247	1416 ± 256	0.038
**Coaptation reserve**			
Leaflet-to-annulus index (LAI)	1.33 ± 0.2	1.28 ± 0.13	0.37
Leaflet area/annulus area	1.22 ± 0.08	1.18 ± 0.07	0.12
(AML + PML)/annulus area (mm/mm^2^)	0.036 ± 0.008	0.031 ± 0.0054	0.07
**Annulus, dynamic**			
Annulus circumference fractional change (%)	5.7 ± 4.7	2.5 ± 5.7	0.046
Annulus area, fractional chance (%)	18 ± 18	7 ± 12	0.035
Early-systolic annulus area (mm^2^)	1134 ± 243	1363 ± 269	0.004
Early-systolic annulus circumference (mm^2^)	127 ± 13	139 ± 13	0.003

AML: anterior mitral leaflet; AP: antero-posterior; EDD: end-diastolic diameter of the left ventricle; EDVi: end-diastolic volume index; EF: ejection fraction of the left ventricle; ESVi: end-systolic volume index; IC: intercommissural; sPAP: systolic pulmonary artery pressure; PML: posterior mitral leaflet.

## Data Availability

The study data will be made available upon request to the corresponding author.
